# Estimation of the severity of breathlessness in the emergency department: a dyspnea score

**DOI:** 10.1186/s12873-017-0125-6

**Published:** 2017-04-26

**Authors:** Tibor Gondos, Viktor Szabó, Ágnes Sárkány, Adrienn Sárkány, Gábor Halász

**Affiliations:** 1 Faculty of Health Sciences, Semmelweis University, Budapest, Hungary; 2 Emergency Department, Jávorszky Ödön Hospital, Vác, Hungary; 3 Department of Hydrodynamic Systems, Budapest University of Technology and Economics, Budapest, Hungary; 4 Emergency Department, “Szent György” University Teaching Hospital, Székesfehérvár, Hungary; 5 Emergency Department, “Kaposi Mór” University Teaching Hospital, Kaposvár, Hungary

**Keywords:** Dyspnea, Emergency, Triage, Score, Blood gas, Lactate, COPD, Heart failure

## Abstract

**Background:**

Dyspnea is a frequent complaint in emergency departments (ED). It has a significant amount of subjective and affective components, therefore the dyspnea scores, based on the patients’ rating, can be ambiguous. Our purpose was to develop and validate a simple scoring system to evaluate the severity of dyspnea in emergency care, based on objectively measured parameters.

**Methods:**

We performed a double center, prospective, observational study including 350 patients who were admitted in EDs with dyspnea. We evaluated the patients’ subjective feeling about dyspnea and applied our Dyspnea Severity Score (DSS), rating the dyspnea in 7 Dimensions from 0 to 3 points. The DSS was validated using the deterioration of pH, base-excess and lactate levels in the blood gas samples (Objective Classification Scale (OCS) 9 points and 13 points groups).

**Results:**

All of the Dimensions correlated closely with the OCS values and with the subjective feeling of the dyspnea. Using multiple linear regression analysis we were able to decrease the numbers of Dimensions from seven to four without causing a significant change in the determination coefficient in any OCS groups. This reduced DSS values (exercise tolerance, cooperation, cyanosis, SpO2 value) showed high sensitivity and specificity to predict the values of OCS groups (the ranges: AUC 0.77–0.99, sensitivity 65–100%, specificity 64–99%). There was a close correlation between the subjective dyspnea scores and the OCS point values (*p* < 0.001), though the scatter was very large.

**Conclusions:**

A new DSS was validated which score is suitable to compare the severity of dyspnea among different patients and different illnesses. The simplified version of the score (its value ≥7 points without correction factors) can be useful at the triage or in pre-hospital care.

## Background

We can define dyspnea as “a subjective experience of breathing discomfort that consists of a quality distinct sensation that varies in intensity” and involves “interactions among multiple physiological, social, and environmental factors, and may induce secondary physiological and behavioral responses” [1, 2]. This symptom is associated with many disorders from psychological problems that are not dangerous to life-threatening conditions. Because it contains a large subjective component its degree does not necessarily correlate well with the severity of the underlying disease, e.g. patients with chronic disease get used to their symptoms and rate their illness much less than the real severity. At the triage in the ED or even in pre-hospital care it is important to estimate the actual severity components of dyspnea in a simple, objective way which reduces reliance on the patient’s subjective feelings.

Several illnesses can cause dyspnea. Dyspnea is only a non-specific sign of these diseases, though in severe form it is a significant warning symptom. The current scoring systems developed to estimate the severity of dyspnea are based mainly on subjective parameters and concentrate only on cardio-pulmonary disorders. The widely used Borg-scale [3] or its modified 10 point version [4] evaluate the patients’ breathlessness from the level of non-existent to the maximum. The effectiveness of this scale has been proved in patients with chronic obstructive pulmonary disease (COPD) or asthma. Mahler and Wells [5] compared three dyspnea rating methods based on the patients’ evaluation and found good correlations with spirometric data in different lung disorders. van der Molen et al. [6] developed a Clinical COPD Questionnaire of 10 items whose effectiveness was proven when compared to the Global Initiative for Chronic Obstructive Lung Disease staging and to the BODE index (body mass index, airflow obstruction, dyspnea, exercise capacity) [7].

Distinguishing between cardiac and pulmonary causes of dyspnea can cause a diagnostic dilemma. Recently, in addition to the patients’ subjective feelings of discomfort, several methods were tested to improve the diagnostic and prognostic efficacy of dyspnea scoring, eg. core-peripheral temperature gradient [8], sequential dyspnea provocation by positioning and walking [9], structured 3-minute walk test [10], S3 captured acoustic cardiography [11], non-invasive measurement of cardiac output and thoracic fluid content [12], including B-type natriuretic peptide levels [13, 14], and even the use of a wide range of biomarkers [15] and physiological variables [16]. The problem with all of these approaches is that they need a specific intervention or tool. Moreover, the process is time-consuming and therefore not suitable for immediate triage decisions. As dyspnea is an important sign of alarm at the triage and because it involves a significant number of subjective and affective components, the patients’ assessment might be misleading. Objective evaluation of the severity of dyspnea is crucial in EDs, but unfortunately, we do not have any single, “magic” parameter which would describe correctly the severity of dyspnea. For objectification, a plausible solution is to choose pH, base excess (BE) and lactate levels in combination, because all of these are easily available in an emergency setting and characterize very well the severity of the patients’ different illnesses [17–25].

With this background, we developed a simple scoring system based on objectively measured parameters that would represent the severity of dyspnea in different illnesses (suitable for scientific comparisons) and help in the immediate decision-making at the triage in the ED or even in pre-hospital care.

## Methods

### Study design and setting

This study was an observational examination using a prospectively collected database analysis conducted in two regional EDs in Hungary (Jávorszky Ödön Hospital, Vác, *n* = 158; “Szent György” University Teaching Hospital, Székesfehérvár, *n* = 192). Informed consent for participation in the study was obtained from every participant. The study was approved by the local Ethical Committees (Institutional Ethical Committee, “Szent György” University Teaching Hospital and Institutional Ethical Committee, Jávorszky Ödön Hospital).

From April 15, 2013 to January 15, 2015 all patients over the age of 18 were recruited who had had any kind of breathing complaints which required a blood gas analysis (venous or arterial sample) with lactate measurement, based on the decision of the examining clinicians. Altogether, 350 patients having complete data at admission were entered into the study. One patient was included only once using the first measurement set in the ED. Those patients who were unable to evaluate their severity of breathlessness were excluded from the study.

### Measurements

After registering the basic demographic data (age, gender, primary reason for emergency admittance) the patients were asked to evaluate their breathlessness using a 10 point numeric scale with 1 point corresponding to the description “I have no breathing problems” and 10 points corresponding to the description “I have severe breathing difficulties, I am almost dead”. After that, the examining physician completed the Dyspnea Severity Score (DSS), rating the dyspnea in 7 dimensions from 0 to 3 points (Table 1). (All of the applied categories are used in daily clinical practice and can represent the severity of dyspnea). The scaling points were arbitrarily determined, with patients being able to earn a maximum score of 21 points.

**Table 1:** Dyspnea Severity Scale

Dimension	Category	0 point	1 point	2 points	3 points
1	Exercise tolerance	No dyspnea during regular activity	Dyspnea during walking	Dyspnea after a few steps	Dyspnea in rest
2	Speech	Undisturbed in rest	Unable to finish a whole sentence	Tells only short sentences or words	Unable to speak, only nodding
3	Cooperation	Executes instructions	Execute instructions after repeated requests	Difficult to tolerate oxygen mask	Agitated, unconsciousness
4	Cyanosis	None	Lips/acro-cyanosis during exercise	Lips/acro-cyanosis in rest	Severe cyanosis with cold sweat
5	SpO2	In rest >95% in ambient air	In rest 90–95% in ambient air, >95% after <5 l/min oxygen inhalation	In rest <90% in ambient air, >95% after high flow of oxygen inhalation	In rest <90% in ambient air, <95% after high flow of oxygen inhalation
6	Breathing	Normal in rest	Respiratory rate >24/min in rest	Using respiratory muscles	Orthopnea
7	Heart rate/rhythm	HR < 100/min in rest	100/min < HR <120/min in rest	120/min < HR <140/min in rest or HR >100/min with arrhythmias	HR >140/min in rest or HR >120/min with arrhythmias

An arterial or a venous blood gas sample was taken from every patient complaining of dyspnea (arterial sampling being preferred when we wanted to know the exact levels of oxygen and carbon dioxide) and pH, BE, and lactate levels were recorded to evaluate the dyspnea in an objective way. All of the parameters were given a point value. Lacking a previous similar analysis, two types of Objective Classification Scale (OCS) were used to estimate the severity of dyspnea. The scaling points were arbitrarily determined, based on clinical practice.

**Fig. 1:**
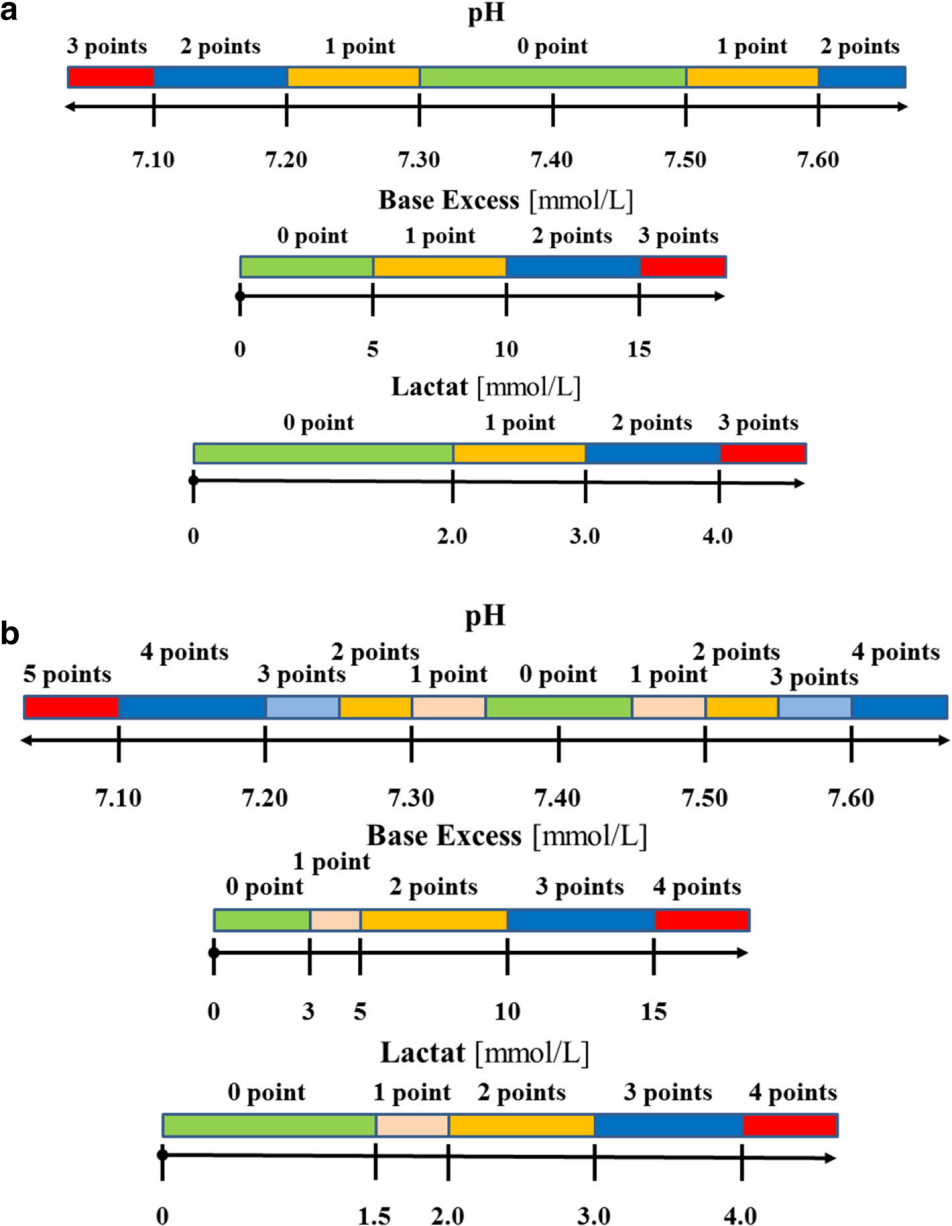
Components of the Objective Classification Scale (**a** 9 points, **b** 13 points)

In the 9 point scale (Fig. 1a) the normal ranges were quite wide, followed by a parallel stepwise increase in severity and OCS points. The maximum score was 9 points.

In the 13 point scale (Fig. 1b) we used narrower normal ranges and put intermediate ranges before the critical. The maximum score was 13 points.

### Data analysis

Data were analyzed using the R Statistics Program, version 3.1.3 [26]. Descriptive statistics included median and interquartile ranges (IQR) for continuous variables and counts and percentages for categorical variables.

Using multivariable regression analysis, OCS values were estimated using the 7 dimensions of the DSS. First, all 7 dimensions were included in the model. Then the numbers of variables were reduced using the forward stepwise method. In the beginning no variables were included in the model. At each step, the variable that improved the most was entered into the analysis until all corresponding regression parameters were not significantly different from zero at *p* < 0.01.

To calculate optimal sensitivity and specificity of the estimated OCS scores, receiver operating characteristics (ROC) curve analysis was performed using the pROC package [27] at different cut-off points. The cut-off point was used to select patients with severe dyspnea based on their original OCS scores both in the 9 and the 13 point system. For example, when the cut-off point was set to 4, all patients with a score of least 4 were assumed to have severe dyspnea. The analysis was performed using the estimated OCS scores with the reduced number of parameters.

To estimate the relationship between the patients’ subjective feelings and objective diagnostic results, subjective scores were compared to estimated dyspnea scores.

## Results

All of the 350 patients included in the study were Caucasian, of whom 199 (56.9%) were female and 151 (43.1%) were male. There was no significant difference between their ages (female: median 73 years, IQR 62, 81 years vs. male: 72 years, IQR 62, 78 years). The primary reasons for admittance were heterogeneous: 184 patients (53%) mainly had pulmonary problems, 152 patients (43%) had cardiac problems, and 34 patients (10%) had other problems which were independent of the cardiopulmonary system. 15 patients had both pulmonary and cardiac problems. The most frequent illnesses were the following: congestive heart failure 137 (39.1%), COPD acute exacerbation 107 (30.6%), pneumonia 62 (17.7%), pulmonary embolism 15 (4.3%), metabolic disease 14 (4%), pulmonary tumor 11 (3.1%), and bronchial asthma, sepsis and acute bronchitis 10-10 cases (2.9%).

The distributions of patients according to the severity of the different dimension scores were quite homogenous for Dimensions 2 (speech), 6 (breathing), 7 (heart rate/rhythm), but asymmetric for Dimension 1 (exercise tolerance) (214 patients - 61.1% - had dyspnea in rest), Dimension 3 (cooperation) (280 patients - 80% - had no problem with communication), and Dimension 4 (cyanosis) (186 patients - 53.1% - had no cyanosis).

Significant correlations were observed for pairs of dimensions (*p* < 0.001). The correlation coefficients changed from 0.228 (Dimension 1 (exercise tolerance) and 4 (cyanosis)) to 0.721 (Dimension 2 (speech) and 6 (breathing)). All of the dimensions also correlated very well with the OCS values and with the subjective experience of the dyspnea, as rated by the patients (Table 2).

**Table 2:** Correlation coefficients between the different Dimensions and the Objective Classification Scales and the subjective rating of the patients

	Dimension 1 (exercise tolerance)	Dimension 2 (speech)	Dimension 3 (coopertation)	Dimension 4 (cyanosis)	Dimension 5 (SpO2)	Dimension 6 (breathing)	Dimension 7 (heart rate/rhythm)
OCS 9 points	0.326	0.440	0.385	0.471	0.417	0.450	0.299
OCS 13 points	0.337	0.398	0.348	0.438	0.409	0.428	0.252
Subjective rating	0.442	0.588	0.419	0.485	0.452	0.615	0.401

To predict the values of the OCSs, multiple linear regression (forward stepping method) was performed using the combination of parameters Dimension 1–7. Increasing the number of dimensions from four to seven did not lead to a significant change in the determination coefficient in any of the OCS groups and showed a very close correlation with the original score values (*r* = 0.988 and *r* = 0.985, respectively). The coefficients, the correlations between the original and estimated OCSs, as well as the determination coefficients (multiple r squared values) of the linear model are presented in Table 3. The determination coefficient which represents the summarized statistical predictive role was better in the OCS 13 point group. Including the patients’ subjective dyspnea rating scores in the analysis did not significantly increase the predictive role of the model.

**Table 3:** Coefficients, correlation and multiple r squared values of the original and the reduced linear models for OCS 9 point and OCS 13 point groups

		Dimension 1 (exercise tolerance)	Dimension 2 (speech)	Dimension 3 (cooperation)	Dimension 4 (cyanosis)	Dimension 5 (SpO2)	Dimension 6 (breathing)	Dimension 7 (heart rate/rhythm)	Correlation coefficients	Multiple r squared
OCS 9 points	Original	0.089	0.117	0.323*	0.418**	0.310	0.211	0.117	0.545	0.628
	Reduced	-	-	0.365*	0.446**	0.418**	0.340**	-	0.541	0.623
OCS 13 points	Original	0.500**	−0.003	0.422	0.582**	0.555**	0.330	0.082	0.550	0.708
	Reduced	0.603**	-	0.522*	0.690**	0.625**	-	-	0.540	0.703

*p < 0.01, and **p < 0.001

**Fig. 2:**
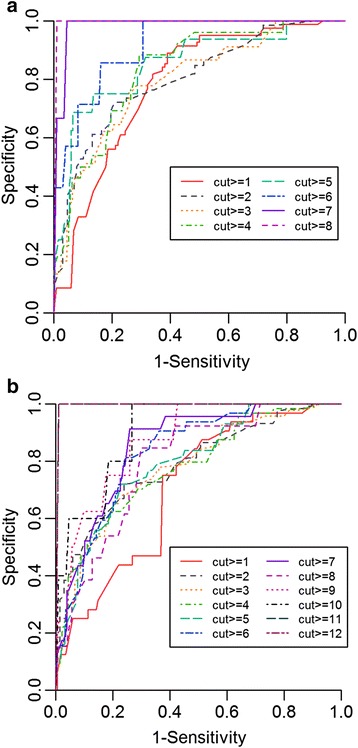
ROC curves for prediction of different levels of OCS values. **a** OCS 9 point group, **b** OCS 13 point group

In order to analyze the sensitivity and specificity of the reduced dimension scales to predict the severity of the OCS values, ROC analysis was performed at different cut-off points. ROC curves for both OCS groups are presented in Fig. 2 with the corresponding tables (Table 4). Increasing the cut-off points resulted in increased AUCs and higher sensitivity and specificity levels.

**Table 4:** Complementary tables for the ROC analysis. “A” OCS 9 point group, “B” OCS 13 point group

OSC point	AUC	Threshold	Sensitivity	Specificity
A				
≥1	0.7701	1.4926	65%	81%
≥2	0.8013	1.6565	76%	75%
≥3	0.7793	1.6565	80%	67%
≥4	0.8012	1.6991	89%	64%
≥5	0.8195	2.75	70%	87%
≥6	0.8485	1.9656	89%	71%
≥7	0.9455	2.7534	100%	83%
≥8	0.9942	4.6219	100%	99%
B				
≥1	0.7544	2.436	76%	71%
≥2	0.7901	3.0616	68%	81%
≥3	0.7822	3.1262	69%	80%
≥4	0.7815	3.1262	76%	72%
≥5	0.7588	3.1262	80%	67%
≥6	0.7986	3.584	86%	68%
≥7	0.7809	3.584	86%	66%
≥8	0.7706	4.3774	71%	78%
≥9	0.7883	4.3692	76%	78%
≥10	0.8694	3.7952	93%	71%
≥11	0.9613	5.0271	100%	84%
≥12	0.9942	7.0638	100%	99%

**Fig. 3:**
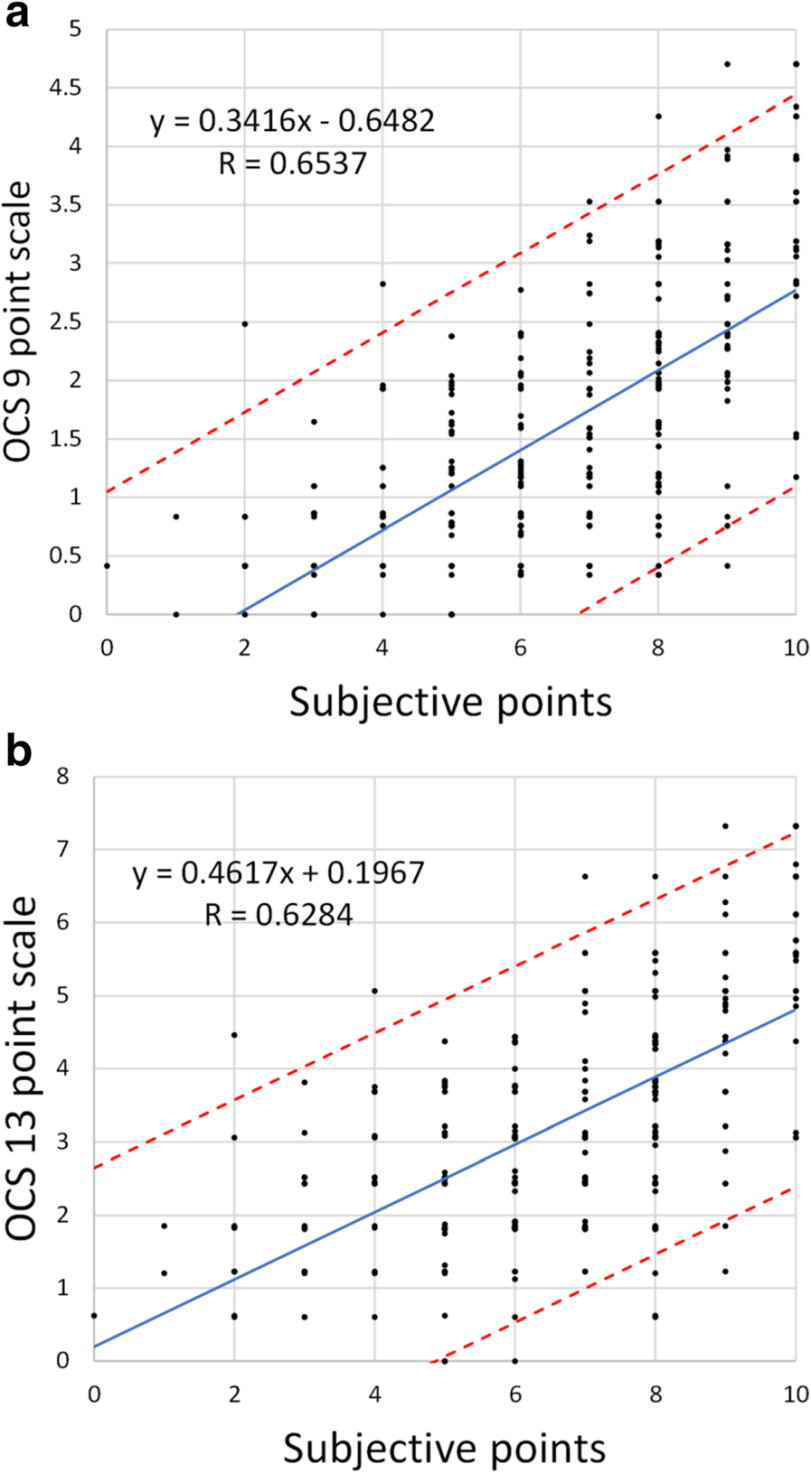
Subjective scores with 95% CI plotted against estimated values of OCSs for the reduced linear model. **a** OCS 9 point group, **b** OCS 13 point group

There was a close correlation (Fig. 3) between the subjective dyspnea rating scores and the reduced OCS 9 point and 13 point values (equivalent to the DSS point values) (*p* < 0.001), though the scatter was very large over the whole range of subjective points.

## Discussion

In this study a simple scoring system was developed that can be utilized regardless of dyspnea etiology or stage of illness, independently of the patient’s subjective evaluation, and this scoring system can also be useful at the emergency triage or even in pre-hospital care. Objective evaluation of the severity of dyspnea is crucial in EDs. Dyspnea is one of the most frequent complaints of the patients admitted and - providing such a distressing signal as it does - may represent the summation of a number of pathophysiological and psychological factors [5]. It is an important sign of alarm at the triage, but because a significant number of subjective and affective components are involved, the patients’ assessment might be misleading.

Unfortunately, we do not have any single, “magic” parameter which would describe correctly the severity of dyspnea. We can improve our diagnostic accuracy using the clinical signs of respiratory distress (tachycardia, tachypnea, abnormal respiratory patterns, cyanosis, nasal flaring, use of accessory respiratory muscles, paradoxical motion of the chest, exercise intolerance, etc.), or their combinations, with different laboratory and other diagnostic testing results (ECG, chest x-ray, CT-scan, echocardiography, etc.) [8–16]. However, these procedures need a significant amount of time and a high level of expert evaluation and financial sourcing. This was the reason we tried in this study to develop a simple scoring system based on objectively measured parameters that would represent the severity of dyspnea in different illnesses and help in the immediate decision-making.

In the first step we developed a severity scale (DSS) including 7 dimensions with the rating possibility from 0 to 3 points. All of the categories were simple to measure and capable of characterizing the severity of dyspnea more objectively than the patients’ feelings of discomfort. These dimensions are also suitable for describing dyspnea independently of the primary cause (pulmonary, cardiac and other forms).

To validate this dyspnea score we compared its value to a more objective rating system (OCS) including certain blood gas parameters and lactate levels. This parameter combination has never been used previously, but as individual parameters they have an important role to play in the evaluation of the patient’s status. The pH and BE values from the blood gas sample and the lactate levels are strongly related in both arterial and venous blood so we can use samples from either origin [28–30]. These three parameters represent a summary of the pathophysiological processes and are independent of our examined categories. The shift of the pH value in any direction means that the compensatory mechanisms of the body in respect of acidosis and alkalosis are exhausted and a significant problem lies behind the dyspnea [31, 32]. Greater changes indicate the presence of more severe underlying diseases. The BE value is a good indication of metabolic compensation [17, 18, 31–34]. Both negative and positive values are warning signs of the severity of illness. Negative values are typical in all kinds of circulatory problems as well as severe metabolic diseases (eg. kidney failure, diabetic ketoacidosis), while positive values occur primarily in the case of COPD patients. Lactate level represents mainly the anaerobic metabolism in the tissues when the patient has no significant liver disease. Increased lactate level correlates very well with the severity of the tissue oxygen metabolism [19–25, 35], independently of the original cause (hypoxia, low blood flow states, oxygen utilization problems in the mitochondria, etc.). Given the lack of previous research, two forms of OCS were used, combining the pH, BE and lactate levels into a score - a 9 point scale and a more detailed 13 point scale.

In our mixed patient population all of the dimension score values showed a significant correlation with either the OCS 9 point or with the OCS 13 point values. This means that the dimensions examined actually represented the severity of dyspnea. Strong correlations were found between the patients’ rating points and the dimension score values, which may also support the adequacy of these dimensions for predicting the severity of dyspnea. Using linear multiple regression analysis to evaluate the summarized role of the dimensions in predicting the OCS point values very strong correlations were found between the original and the estimated scores. The best result was in the OCS 13 point model where the multiple r squared value was 0.708. Surprisingly, including the patients’ dyspnea score values in the analysis only caused minimal changes in the prediction. This means that the dimensions relate more closely to the objective dyspnea markers than the patients’ subjective ratings. Linear regression analysis of patients’ rating score values and the decreased dimension scores (Fig. 3) also demonstrate that patients’ subjective feelings are not in accordance with the objective assessment of dyspnea.

Using forward stepwise model of multiple regression analysis we were able to reduce the number of dimensions from seven to four parameters without increasing the prediction error significantly. The best result was found in the OCS 13 point model where, by including Dimension 1, 3, 4 and 5, the prognostic probability did not decrease significantly compared to the original model where all of the dimensions were included. The high correlation coefficients in the linear regression analysis (Table 3) also reinforced the conclusion that only four parameters were necessary to predict the OCS values.

To compare the applicability of the Four Dimension Model for categorizing the severity of dyspnea, ROC analysis was performed for different cut-off points. The optimal cut-off point was ≥4 points for the OCS 9 point model (sensitivity: 89%, specificity: 64%, AUC: 0.8021) and ≥7 points for the OCS 13 point model (sensitivity: 86%, specificity: 68%, AUC: 0.7809). However, values of AUC ranging between 0.77 and 0.99 for OCS 9 points and 0.75 and 0.99 for OCS 13 points suggest that the chosen parameters can be used to detect dyspnea in this mixed emergency care population at a wide range of cut-off points.

### Limits of the study

This study has some limitations. First, the number patients included was enough to analyze their data as a whole but it was insufficient to make a detailed evaluation in respect of age, gender, and basic illnesses. Second, the validation based on blood gas parameters and lactate level taken from arterial or venous blood was not evidence based. According to the result of recent articles [28–30, 36] venous and arterial pH and bicarbonate agree reasonably well at all values and the lactate level showed a poorer agreement only at abnormal values. This was the scientific background using arterial or venous samples. In a few cases during the study, a parallel sampling resulted in a close correlation between pH, BE and lactate levels but this analysis has not been published yet. Third, we did not collect outcome data from the subsequent progress of the patients, so the developed dyspnea score is validated only in an emergency triage situation. And finally, we did not compare our data with other scoring systems to evaluate which one is more effective in predicting dyspnea severity.

## Conclusion

In summary, we have developed a new, simple dyspnea scoring system derived from four dimensions (exercise tolerance, cooperation, cyanosis, SpO2 value; multiplied by appropriate coefficients), which correlates well with objective classification parameters. The simplified version of the score (its value ≥7 points without correction factors) can be useful at the triage or in pre-hospital care.

## Abbreviations

AUC: Area under the curve
BE: Base excess
CI: Confidence interval
COPD: Chronic obstructive pulmonary disease
DSS: Dyspnea Severity Score
ED: Emergency department
HR: Heart rate
IQR: Interquartile ranges
OCS: Objective Classification Scale
ROC: Receiver operating characteristics
SpO2: Pulzoxymetric oxygen saturation

## References

[1] American Thoracic Society (1999). Dyspnea. Mechanisms, assessment, and management: a consensus statement. Am J Respir Crit Care Med.

[2] Parshall MB, Schwartzstein RM, Adams L (2012). An official ATS statement: update on the mechanisms, assessment, and management of dyspnea. Am J Respir Crit Care Med.

[3] Borg G (1982). Psychophysical bases of perceived exertion. Med Sci Sports Exerc.

[4] Kendrick KR, Baxi SC, Smith RM (2000). Usefulness of the modified 0-10 Borg scale in assessing the degree of dyspnea in patients with COPD and asthma. J Emerg Nurs.

[5] Mahler DA, Wells CK (1988). Evaluation of clinical methods for rating dyspnea. Chest.

[6] van der Molen T, Willemse BWM, Schokker S (2003). Development, validity and responsiveness of the clinical COPD questionnaire. Health Qual Life Outcomes.

[7] Liu S-F, Tseng C-W, Tu M-L, Wang CC (2012). The clinical COPD questionnaire correlated with BODE index - a cross-sectional study. Scient World J.

[8] Clarke SF, Parris RJ, Reynard K (2005). Core-peripheral temperature gradient as a diagnostic test in dyspnea. Emerg Med J.

[9] Pang PS, Cleland JGF, Teerlink JR, for the Acute Heart Failure Syndromes International Working Group (2008). A proposal to standardize dyspnea measurement in clinical trials of acute heart failure syndromes: the need for a uniform approach. Eur Heart J.

[10] Pan AM, Stiell IG, Clement CM, Acheson J, Aaron SD (2009). Feasibility of a structured 3-minute walk test as a clinical decision tool for patients presenting to the emergency department with acute dyspnea. Emerg Med J.

[11] Collins SP, Peacock WF, Lindsell CJ (2009). S3 detection as a diagnostic and prognostic aid in emergency department patients with acute dyspnea. Ann Emerg Med.

[12] García X, Simon P, Guyette FX (2013). Noninvasive assessment of acute dyspnea in the ED. Chest.

[13] Moe GW, Howlett J, Januzzi JL, for the Canadian Multicenter Improved Management of Patient With Congestive Heart Failure (IMPROVE-CHF) Study Investigators (2007). N-terminal B-type natriuretic peptide testing improves the management of patients with suspected acute heart failure. Preliminary results of the Canadian prospective randomized multicenter IMPROVE-CHF study. Circulation.

[14] Jang TB, Aubin C, Naunheim R, Lewis LM, Kaji AH (2012). The predictive value of physical examination findings in patients with suspected acute heart failure syndrome. Intern Emerg Med.

[15] Eurlings LW, van Wijk SS, van Kimmenade R (2012). Multimarker strategy for short-term risk assessment in patients with dyspnea in the Emergency Department. The MARKED (Multi mARKer Emergency Dyspnea)-Risk Score. J Am Coll Cardiol.

[16] Baggish AL, Lloyd-Jones DM, Blatt J (2008). A clinical and biochemical score for mortality prediction in patients with acute dyspnoea: derivation, validation and incorporation into a bedside programme. Heart.

[17] Ibrahim I, Chor WP, Chue KM (2016). Is arterial base deficit still a useful prognostic marker in trauma? A systematic review. Am J Emerg Med.

[18] Lam SW, Lingsma HF, van Beek EDF, Leenem PLH (2016). Validation of a base deficit-based trauma prediction model and comparison with TRISS and ASCOT. Eur J Trauma Emerg Surg.

[19] Pedersen M, Brandt VS, Holler JG, Lassen AT (2015). Lactate level, aetiology and mortality of adult patients in an emergency department: a cohort study. Emerg Med J.

[20] El-Kersh K, Chadda U, Sinha RS (2015). Predictive role of admission lactate level in critically ill patients with acute gastrointestinal bleeding. J Emerg Med.

[21] Gwak MH, Jo S, Jeong T (2015). Initial serum lactate level is associated with inpatient mortality in patients with community-acquired pneumonia. Am J Emerg Med.

[22] Chen Y-X, Li C-S (2015). Lactate on emergency department arrival as a predictor of mortality and site-of-care in pneumonia patients: a cohort study. Thorax.

[23] Vanni S, Jimenez D, Nazerian P (2015). Short-term clinical outcome of normotensive patients with acute PE and high plasma lactate. Thorax.

[24] Barfod C, Lundstrom H, Lauritzen MMP (2015). Peripheral venous lactate at admission is associated with in-hospital mortality, a prospective cohort study. Acta Anaesthesiol Scand.

[25] Datta D, Walker C, Gray AJ (2015). Arterial lactate levels in an emergency department are associated with mortality: a prospective observational cohort study. Emerg Med J.

[26] R Core Team. R: A language and environment for statistical computing. Vienna: R Foundation for Statistical Computing; 2016. https://www.R-project.org/.

[27] Robin X, Turck N, Hainard A (2011). pROC: an open-source package for R and S+ to analyze and compare ROC curves. BMC Bioinformatics.

[28] Zakrison T, McFarlan A, Wu YY (2013). Venous and arterial base deficits: Do these agree in occult shock and in the elderly? A Bland-Altman analysis. J Trauma Acute Care Surg.

[29] Kelly A-M, Klim S. Agreement between arterial and venous pH and pCO2 in patients undergoing non-invasive ventilation in the emergency department. Emerg Med Australasia. 2013;25:203–6. 10.1111/1742-6723.12066 23759038

[30] Goyal N, Taylor AR, Rivers EP. Relationship between central and peripheral venous oxygen saturation and lactate levels: a prospective study. J Emerg Med. 2016;50:809–17. 10.1016/j.jemermed.2016.03.021 27210904

[31] Adrogué HJ, Madias NE (1998). Management of life-threatening acid-base disorders. N Eng J Med.

[32] Kaplan LJ, Frangos S (2005). Clinical review: Acid–base abnormalities in the intensive care unit. Crit Care.

[33] Frantz TL, Gaski GE, Terry C (2016). The effect of pH versus base deficit on organ failure in trauma patients. J Surg Res.

[34] Mutschler M, Nienaber U, Wafaisade A (2014). The impact of severe traumatic brain injury on a novel base deficit- based classification of hypovolemic shock. Scand J Trauma Resusc Emerg Med.

[35] Allen M (2011). Lactate and acid base as a hemodynamic monitor and markers of cellular perfusion. Pediatr Crit Care Med.

[36] Bloom BM, Grundlingh J, Bestwick JP, Harris T (2014). The role of venous blood gas in the Emergency Department: a systematic review and meta-analysis. Eur J Emerg Med.

